# Prognostic Accuracy of Screening Tools for Clinical Deterioration in Adults With Suspected Sepsis in Northeastern Thailand: A Cohort Validation Study

**DOI:** 10.1093/ofid/ofae245

**Published:** 2024-05-02

**Authors:** Jenna Wixon-Genack, Shelton W Wright, Natalie L Cobb Ortega, Viriya Hantrakun, Kristina E Rudd, Prapit Teparrukkul, Direk Limmathurotsakul, T Eoin West

**Affiliations:** Department of Internal Medicine, Alaska Native Medical Center, Anchorage, Alaska, USA; Division of Pediatric Critical Care Medicine, Department of Pediatrics, University of Washington, Seattle, Washington, USA; Division of Pulmonary, Critical Care and Sleep Medicine, Department of Medicine, University of Washington, Seattle, Washington, USA; Mahidol-Oxford Tropical Medicine Research Unit, Faculty of Tropical Medicine, Mahidol University, Bangkok, Thailand; Department of Critical Care Medicine, University of Pittsburgh, Pittsburgh, Pennsylvania, USA; Department of Internal Medicine, Sunpasitthiprasong Hospital, Ubon Ratchathani, Thailand; Mahidol-Oxford Tropical Medicine Research Unit, Faculty of Tropical Medicine, Mahidol University, Bangkok, Thailand; Department of Tropical Hygiene, Faculty of Tropical Medicine, Mahidol University, Bangkok, Thailand; Division of Pulmonary, Critical Care and Sleep Medicine, Department of Medicine, University of Washington, Seattle, Washington, USA

**Keywords:** early warning systems, low-and middle-income countries, NEWS, sepsis, Southeast Asia

## Abstract

**Background:**

We sought to assess the performance of commonly used clinical scoring systems to predict imminent clinical deterioration in patients hospitalized with suspected infection in rural Thailand.

**Methods:**

Patients with suspected infection were prospectively enrolled within 24 hours of admission to a referral hospital in northeastern Thailand between 2013 and 2017. In patients not requiring intensive medical interventions, multiple enrollment scores were calculated including the National Early Warning Score (NEWS), the Modified Early Warning Score, Between the Flags, and the quick Sequential Organ Failure Assessment score. Scores were tested for predictive accuracy of clinical deterioration, defined as a new requirement of mechanical ventilation, vasoactive medications, intensive care unit admission, and/or death approximately 1 day after enrollment. The association of each score with clinical deterioration was evaluated by means of logistic regression, and discrimination was assessed by generating area under the receiver operating characteristic curve.

**Results:**

Of 4989 enrolled patients, 2680 met criteria for secondary analysis, and 100 of 2680 (4%) experienced clinical deterioration within 1 day after enrollment. NEWS had the highest discrimination for predicting clinical deterioration (area under the receiver operating characteristic curve, 0.78 [95% confidence interval, .74–.83]) compared with the Modified Early Warning Score (0.67 [.63–.73]; *P* < .001), quick Sequential Organ Failure Assessment (0.65 [.60–.70]; *P* < .001), and Between the Flags (0.69 [.64–.75]; *P* < .001). NEWS ≥5 yielded optimal sensitivity and specificity for clinical deterioration prediction.

**Conclusions:**

In patients hospitalized with suspected infection in a resource-limited setting in Southeast Asia, NEWS can identify patients at risk of imminent clinical deterioration with greater accuracy than other clinical scoring systems.

## Teaser

In a cohort study of nearly 2700 prospectively enrolled patients with suspected sepsis in rural Southeast Asia, the National Early Warning Score has superior prediction of imminent clinical deterioration compared with other frequently used screening tools.

Sepsis, defined as organ dysfunction from infection-related immune dysregulation, is a major cause of global disease and death [1]. Critically, sepsis disproportionately impacts low- and middle-income countries [2]. In tropical Southeast Asia, infection-related hospitalization is common, often progresses to sepsis, and infectious causes are diverse [3].

To rapidly identify hospitalized patients at high risk of clinical deterioration, multiple early warning scores have been developed and are commonly used in healthcare centers in North America, Europe, and Australia; scoring systems include the National Early Warning Score (NEWS) and the Modified Early Warning Score (MEWS) [4, 5]. Another early warning system, Between the Flags (BTF), has been developed for hospitalized patients in Australia [6, 7]. The quick Sequential Organ Failure Assessment (qSOFA) score, requiring only 3 clinical examination components, was initially developed to specifically help clinicians identify hospitalized patients at risk of sepsis outside of an intensive care setting [8]. Although NEWS and MEWS were not originally designed to identify patients at high risk of progression to sepsis, they have been frequently used as sepsis screening tools in resource-rich settings, and 2021 Surviving Sepsis guidelines recommended against using qSOFA over NEWS or MEWS to screen for sepsis [9, 10].

In patients with suspected infection in the United States, multiple studies suggest that NEWS may be superior for predicting death or intensive care unit (ICU) transfer compared with other commonly used scores [11, 12]. However, established early warning scores may face limitations in resource-constrained regions, such as barriers to implementation, unique risk factors for sepsis-related outcomes, and low sepsis awareness despite a high burden of disease [1, 2, 13, 14]. For example, a study in Malawi reported reduced sensitivity and specificity of MEWS for predicting early death in hospitalized patients [15]. In addition, the Royal College of Physicians in the United Kingdom initially developed NEWS to identify patients at risk of imminent clinical deterioration, typically within 24 hours of score calculation, though this outcome is not commonly assessed [5, 16, 17]. Therefore, we sought to validate the accuracy of NEWS for predicting imminent clinical deterioration in patients hospitalized with suspected infection in northeastern Thailand and to compare its performance with that of other commonly used clinical assessment tools. To our knowledge, this is the largest prospective study to assess the prediction accuracy of clinical warning scores for early deterioration in a resource-constrained setting.

## METHODS

### Study Design and Participants

Patients aged ≥18 years admitted to Sunpasitthiprasong Hospital in Ubon Ratchathani, Thailand with suspected sepsis were prospectively enrolled between 2013 through 2017. This cohort has been described elsewhere [18]. In brief, patients admitted with suspected or documented infection within the prior 24 hours were eligible. In addition, patients were required to have ≥3 of 20 systemic manifestations of infection proposed as diagnostic criteria for sepsis by the 2012 Surviving Sepsis Campaign (Supplementary Table 1) [19]. Recruitment occurred through screening medical records of patients admitted to the emergency department, medical wards and medical ICUs. Assessments by the study team, including collection of clinical and laboratory data, occurred at the time of enrollment as well as on the subsequent calendar days. All enrolled patients were contacted 28 days after enrollment to determine 28-day mortality and date of death, if applicable. In this secondary analysis, patients not requiring mechanical ventilation, vasoactive medications, or ICU admission at the time of enrollment were subsequently selected for inclusion in the analysis cohort.

### Clinical Definitions

Four scores (NEWS, MEWS, qSOFA, and BTF) were calculated using clinical data available closest to the time of enrollment. Each score components, relevant variables and availability of data are listed in Supplementary Tables 2 and 3. A Glasgow Coma Scale (GCS) was calculated at the time of enrollment by the study team. As some of the calculated scores use an AVPU (alert, voice, pain, unresponsive) rating rather than GCS, a GCS ≤13 was considered equivalent to a V, P, or U rating for NEWS calculation. For MEWS and BTF calculations, a GCS ≤8 was considered equivalent to a P/U rating and a GCS of 9–13 was considered equivalent to a V rating [20]. To convert BTF to a numerical score for model development, patients meeting any yellow zone criteria were assigned 1 point and those meeting any red zone criteria were assigned 2 points [11, 21]. Patients not meeting either zone's criteria were assigned a 0 and classified in a “low-risk” category. For consistency with previous methods, when score components were not available, 0 points were assigned [8, 22].

### Outcome Measure

The primary outcome measure was imminent clinical deterioration, defined as the new requirement of mechanical ventilation, vasoactive medications, ICU admission, or death within 1 day of enrollment. NEWS originally defined imminent clinical deterioration within 24 hours after score calculation [5]. In our original study, follow-up data were collected prospectively by the study team each calendar day after patient enrollment. To minimize the effect of variable follow-up times in this secondary analysis, only patients with follow-up data obtained during a window of 20–28 hours following enrollment were included. At the study hospital, it is common practice to rapidly discharge both improving patients as well as those in moribund conditions who wish to die at home. In this secondary analysis, patients discharged before the follow-up window were included in the analysis and were considered to meet the outcome criteria if they died within 1 calendar day of enrollment. However, data regarding the other elements of the imminent clinical deterioration definition were not available for patients discharged before the follow-up assessment.

### Statistical Analysis

Enrollment clinical data, including calculated scores, were summarized using proportions for discrete variables and medians and interquartile ranges for continuous variables. The association of each score with clinical deterioration was evaluated by logistic regression. Discrimination for predicting clinical deterioration was subsequently evaluated by generating the area under the receiver operating characteristic curve (AUROC). Models were assessed for bias by internal validation using 10-fold cross-validation [23]. Comparisons of AUROCs were made using the roccomp command in Stata software. Cutoff values representing optimal discrimination were determined using Youden's index [24]. Subsequently, the sensitivity, specificity, and negative and positive predictive likelihood ratios with corresponding 95% confidence intervals were calculated. Analyses were performed using Stata/SE software, version 14.2. Reporting guidelines for multivariable prediction model validation were followed [25].

### Sensitivity Analyses

Multiple sensitivity analyses were performed. In our primary analysis, missing variables were assumed to be normal during score calculation [8, 22]. Two sensitivity analyses were therefore also performed related to bias from this treatment of missing data: (1) a complete case analysis including only patients without any missing variables and (2) an analysis in which the most recent available prior value was used when a variable was missing, similar to prior methods [12]. In addition, patients were often enrolled near the time of hospital admission, and all were enrolled within 24 hours of admission. Therefore, a third sensitivity analysis using the most abnormal values between admission or enrollment was performed, though early warning scores may not be used this way in clinical practice. Finally, as this was a secondary analysis of a prospectively collected data set, the exact follow-up times for enrolled patients was variable. To minimize variability in the time between enrollment score calculation and the outcome measurement, a 24 ± 4-hour window was selected a priori. However, to account for the possibility that excluded patients with follow-up outside this window may be relevant to score prediction accuracy, we performed a fourth sensitivity analysis for follow-up data available during a 24 ± 8-hour window after the time of enrollment.

### Patient Consent Statement

Written informed consent was obtained from study participants or their representatives before enrollment. The studies were approved by the Sunpasitthiprasong Hospital Ethics Committee (no. 039/2556), the Ethics Committee of the Faculty of Tropical Medicine, Mahidol University (no. MUTM2012-024-01) the University of Washington Institutional Review Board (no. 42988). and the Oxford University Tropical Research Ethics Committee (no. OXTREC172-12).

## RESULTS

### Patient Characteristics

**Figure 1.**
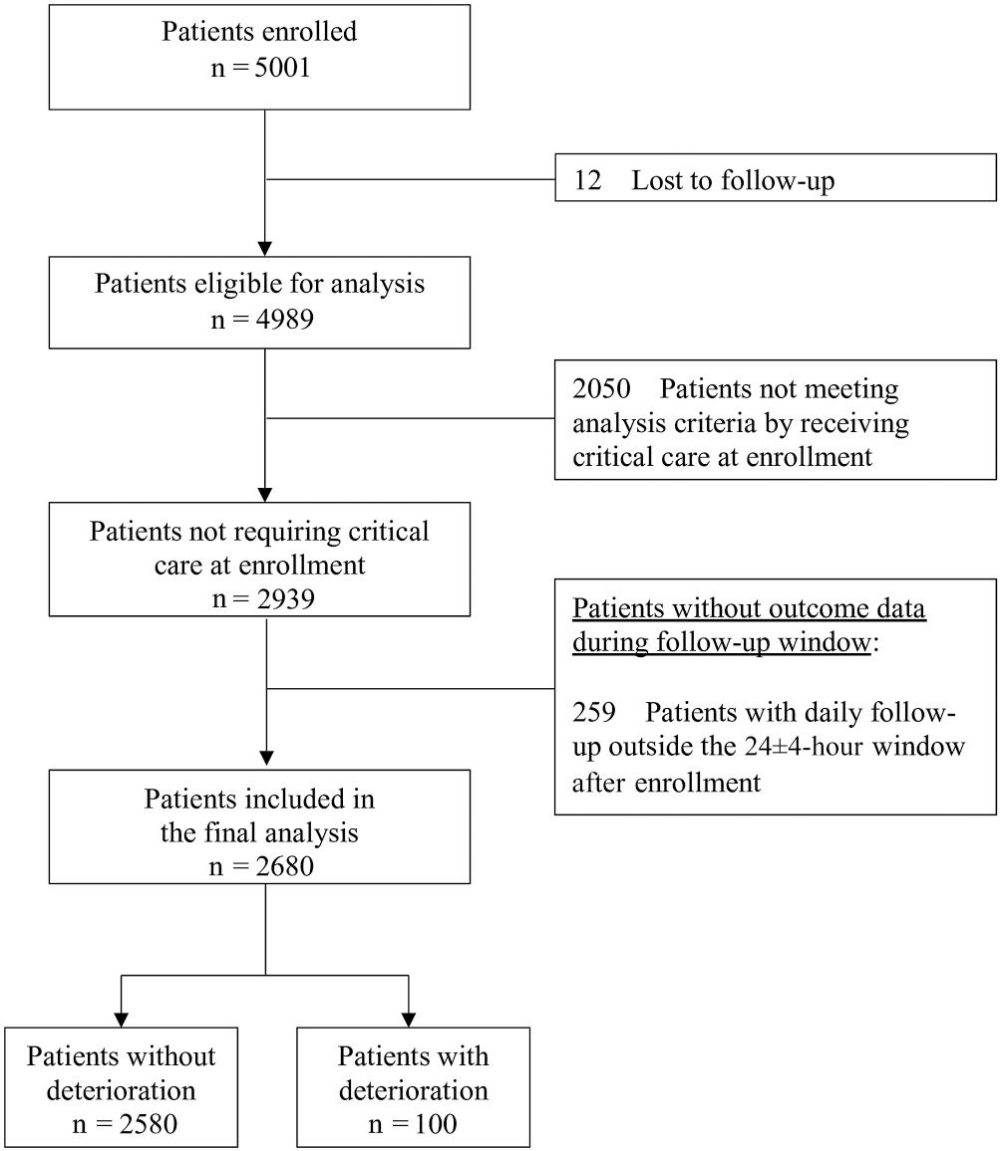
Study flow chart, showing the analysis of the cohort.

Of 4989 patients in the original cohort, 2939 were not admitted to the ICU or required mechanical ventilation or vasoactive medications at enrollment and were retained for this secondary analysis. Of this selected cohort, 2680 had follow-up data within 24 ± 4 hours after enrollment (Figure 1); 259 were excluded from analysis because their follow-up occurred outside the 24 ± 4-hour window. Of the 2680 patients in the final analysis cohort, 138 (5%) were discharged before the follow-up period. The clinical characteristics of the final analysis cohort are listed in Table 1. The median age in this cohort (interquartile range) was 53 (34–68) years, and 49% identified as female. In this cohort, 1661 of 2680 (62%) were referred from 53 different hospitals in the region; 100 of the 2639 patients (4%) met the criteria of clinical deterioration within a day (24 ± 4 hours) of enrollment (outcome distribution provided in Supplementary Figure 1).

**Table 1.** Patient Characteristics

Characteristic	Patients, No. (%)^a^ (n = 2680)
Demographics	
Age, median (IQR), y	53 (34–68)
** ** Female sex by self-report	1302 (49)
Preexisting conditions	
** ** Charlson Comorbidity Index, median (IQR)	1 (0–3)
** ** Diabetes	489 (18)
** ** Chronic liver disease	65 (2)
** ** Chronic kidney disease	262 (10)
** ** Chronic cardiovascular disease	113 (4)
** ** Chronic lung disease	175 (7)
** ** Cancer	38 (1)
** ** HIV	36 (1)
Referred from another facility	1661 (62)
Originating hospitals, no.	53
Clinical scores	
** ** NEWS, median (IQR)	4 (3–6)
** ** MEWS, median (IQR)	2 (1–3)
** ** qSOFA score, median (IQR)	1 (0–1)
** ** BTF	…
** ** Low risk	1378 (51)
** ** Yellow zone	1013 (38)
** ** Red zone	289 (11)
Outcome	
Clinical deterioration	100 (4)
Mechanical ventilation^b^	43 (43)
Vasoactive requirement^b^	38 (38)
** ** ICU admission^b^	33 (33)
** ** Death^b^	18 (18)

Abbreviations: BTF, Between the Flags; HIV, human immunodeficiency virus; ICU, intensive care unit; IQR, interquartile range; MEWS, Modified Early Warning Score; NEWS, National Early Warning Score; qSOFA, quick Sequential Organ Failure Assessment.

^a^Data represent no. (%) of patients unless otherwise specified.

^b^Percentage listed for each clinical deterioration component is the percentage of patients with clinical deterioration.

Clinical scores were calculated at the time of enrollment, and summarized scores are listed in Table 1. The distribution of each score and related proportion of clinical deterioration was then calculated for each score level. For each of the 4 scores, the proportion of patients experiencing a clinical deterioration generally increased with higher scores (Supplementary Figure 2).

**Figure 2.**
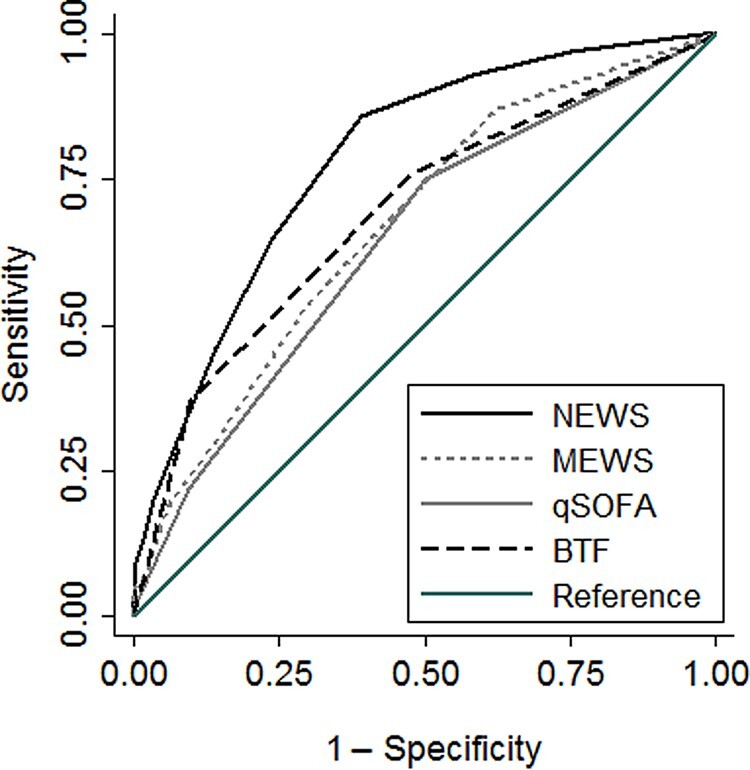
Area under the receiver operating characteristic curves for predicting clinical deterioration, comparing the National Early Warning Score (NEWS), the Modified Early Warning Score (MEWS), the quick Sequential Organ Failure Assessment (qSOFA) score, and Between the Flags (BTF).

We next determined whether an incremental increase in each score increased the odds of clinical deterioration. Each of the 4 scores was significantly associated with clinical deterioration (odds ratios [95% confidence intervals (CIs)] as follows: NEWS, 1.5 [1.4–1.7]; MEWS, 1.5 [1.4–1.7]; qSOFA, 2.2 [1.7–2.9]; BTF, 2.9 [2.2–3.9]; all *P* < .001). We next measured the ability of each score to discriminate clinical deterioration prediction. For this analysis, NEWS was selected a priori as the standard for comparison. Among the calculated scores, NEWS had significantly higher discrimination for predicting clinical deterioration (AUROC, 0.78 [95% CI, .74–.83]) compared with MEWS (0.67 [.63–.73]), qSOFA (0.65, [.60–.70]), and BTF (0.69 [.64–.75]) (all *P* < .001) (Table 2 and Figure 2).

**Table 2.** Discrimination of Clinical Scores for Predicting Clinical Deterioration

Model	Cohort AUROC (95% CI)	*P* Value	Cross-validation AUROC (95% CI)^a^
NEWS	0.78 (.74–.83)	Reference	0.77 (.73–.82)
MEWS	0.67 (.63–.73)	<.0001	0.66 (.61–.71)
qSOFA	0.65 (.60–.70)	<.0001	0.62 (.56–.67)
BTF	0.69 (.64–.75)	<.001	0.66 (.60–.72)

Abbreviations: AUROC, area under the receiver operating characteristic curve; BTF, Between the Flags; CI, confidence interval; MEWS, Modified Early Warning Score; NEWS, National Early Warning Score; qSOFA, quick Sequential Organ Failure Assessment.

^a^Ten-fold internal cross-validation.

We also performed multiple sensitivity analyses: (1) a complete case analysis in 2103 patients with complete variable data at the time of enrollment, (2) an analysis calculating scores using the most recent prior value when a score variable was missing, (3) an analysis calculating the worst score variable within the time from admission to enrollment, and (4) an analysis using enrollment scores but including 2930 patients with follow-up data available during a 24 ± 8-hour window after enrollment. Relative discrimination of clinical deterioration remained similar among all assessed scores (Supplementary Table 4).

Because of the relative strength of NEWS compared with the other assessed scores, we calculated an optimal NEWS threshold using a Youden index and determined the subsequent clinical performance of this threshold in our cohort. An optimal NEWS of ≥5 had a sensitivity of 89% (95% CI, 81%–94%) and specificity of 54% (52%–56%) for clinical deterioration. A NEWS of ≥6 decreased sensitivity to 76% (95% CI, 66%–84%) but improved specificity to 70% (68%–72%). Complete clinical performance characteristics of these NEWS thresholds are listed in Table 3.

**Table 3.** Performance of National Early Warning Score Thresholds for Clinical Deterioration Prediction

Measure (95% CI)	NEWS ≥5	NEWS ≥6
Sensitivity	**89 (81–94)** (n = 89/100)	**76 (66–84)** (n = 76/100)
Specificity	**54 (52–56)** (n = 1384/2580)	**70 (68–72)** (n = 1806/2580)
Positive predictive value	**7 (6–9)** (n = 89/1285)	**9 (7–11)** (n = 76/774)
Negative predictive value	**99 (99–100)** (n = 1384/1395)	**99 (98–99)** (n = 1963/1998)
Positive likelihood ratio	**1.9 (1.8–2.1)**	**2.5 (2.2–2.9)**
Negative likelihood ratio	**0.2 (0.1–0.4)**	**0.3 (0.2–0.5)**

Abbreviations: CI, confidence interval; NEWS, National Early Warning Score.

The bold values are defined by the Measure (95% CI).

## DISCUSSION

In a large, prospectively enrolled cohort of patients hospitalized with suspected infection in northeastern Thailand, NEWS was superior to 3 other scoring systems for predicting early clinical deterioration. To our knowledge, this study is the largest prospective validation of NEWS undertaken to date in a resource-constrained setting.

NEWS and its successor, NEWS-2, were originally developed in the United Kingdom as simple scoring systems to alert healthcare providers to hospitalized patients with a high risk of clinical decline outside of ICUs [16, 17]. Multiple large studies in resource-rich settings have demonstrated that NEWS can outperform other scores in predicting clinical decline. An analysis of electronic health record data from a large healthcare system in the United States found that NEWS was superior to MEWS, qSOFA, systemic inflammatory response syndrome (SIRS), and BTF at predicting ICU admission or death during hospitalization in patients with or without infection [11]. A similar US-based study of hospitalized patients with suspected infection also reported that NEWS performed better than several other scores at identifying patients outside the ICU at risk of in-hospital death or ICU transfer [12].

However, the original evaluations of NEWS focused on its prediction of imminent clinical deterioration, typically within 24 hours after score calculation, and not longer-term outcomes [5, 16]. Evaluations of NEWS in resource-constrained settings have been similarly limited by retrospective study design or identification of individuals at risk of different outcomes, such as in-hospital death or sepsis diagnosis [26, 27]. In our study, we specifically sought to validate the performance of NEWS in identifying patients at highest risk of imminent clinical deterioration in a large prospectively recruited cohort of patients with suspected infection in northeastern Thailand.

Whether specific scoring systems may be superior in identifying high-risk patients with suspected infection is a matter of debate. qSOFA, for example, was not originally designed as an early warning score but rather to identify patients at risk of sepsis outside of a critical care setting, and it has been implemented as a tool for early sepsis identification in resource-limited settings [8, 28]. Indeed, the 2021 Surviving Sepsis Guidelines recommended against using qSOFA over other scores as single screen for sepsis [10]. Nevertheless, due to its simplicity, qSOFA remains an attractive tool for resource-limited settings. We have also reported that augmenting the qSOFA score with a point-of-care lactate concentration may have similar 28-day outcome prediction in Thai patients with suspected infection as the more cumbersome Sequential Organ Failure Assessment (SOFA) [29]. However, in this study, qSOFA had significantly lower accuracy than NEWS for predicting imminent clinical deterioration. MEWS, which uses a method similar to that of NEWS, though without oxygen-related data, has strong prediction of infection-related outcomes in high-resource settings but had markedly worse predictive accuracy of clinical deterioration compared with NEWS in our study [12].

In our cohort, the optimal NEWS threshold was 5, the same threshold for activating an urgent healthcare provider response proposed by the Royal College of Physicians [16]. This similar target across diverse patient populations possibly highlights the strength of a more comprehensive clinical score [19]. However, a more comprehensive scoring system may also not be practical in a resource-limited setting. For example, per the Royal College of Physicians, a NEWS of ≥5 activates an escalation protocol including hourly assessments along with an urgent clinician assessment [17]. While our study suggests that a patient with a NEWS of ≥5 may be at higher risk of deterioration in the next 24 hours, whether an urgent healthcare provider response may be beneficial is unknown, particularly if additional interventions are limited.

While the close monitoring recommended by the Royal College of Physicians may pose challenges in a resource-limited setting, implementation of NEWS may improve patient monitoring and outcomes, even when protocol adherence is poor [30]. In addition, a NEWS threshold of ≥5 in our study had a reasonably high sensitivity of 89% and a negative predictive value of 99%, suggesting that intermittent screening may have value in identifying which patients are at lower risk of deterioration, allowing for triage and resource utilization. However, further research is necessary to understand the utility and potential implementation challenges of NEWS in resource-limited settings.

Our study has several strengths. Data in the parent study were collected prospectively, a rarity in the region. Enrollment was undertaken within 24 hours after admission, and for this study we analyzed patients with dedicated follow-up occurring 20–28 hours after enrollment. This study design resulted in minimal missing data and rigorous capture of cases with early clinical deterioration. Our broad definition of clinical deterioration did not require ICU transfer, as this may not be appropriate in resource-limited settings where critical care interventions are often provided outside of a dedicated ICU facility [22, 31]. In addition, the parent study was performed in northeast Thailand, where infectious causes are diverse [3, 32]. Finally, we performed numerous sensitivity analyses to account for variability in prospective data collection and patient inclusion.

Our study also has several limitations. Each of the assessed clinical scoring systems was developed in resource-rich settings and may have limitations in its implementation in other settings [13, 14]. This was a single-center study of a referral hospital in northeast Thailand. Although 62% of the cohort was transferred from 53 different hospitals—representing a broad catchment of the region—our results may not be representative of other settings or the referring healthcare centers. As study enrollment began in 2013, patients were identified as having suspected sepsis using contemporary criteria and not using current sepsis guidelines, though these may not be appropriate for resource-limited settings [33, 34]. We also compared 4 scores widely studied across economic settings, but other novel scores, including those localized to our specific setting, may have superior performance.

The clinical data used to calculate scores were collected prospectively at the time of enrollment, but the predictive performance of sequential scores or scores calculated later in the hospital stay—or prediction of deterioration beyond 24 hours after score calculation—is unknown. Furthermore, how these scores would perform outside of a prospective study may be different [35]. Finally, we did not evaluate the performance of NEWS-2 [17]. While NEWS-2 may have additional strengths compared with NEWS, it requires the additional consideration of values for the arterial partial pressure of carbon dioxide. In our study, arterial blood gas measurements were not frequently obtained outside the ICU, limiting calculation of NEWS-2. However, this may reflect the advantages of a score based on readily available clinical data points in resource-limited settings.

In conclusion, we report that NEWS is superior to other early warning scores and to qSOFA in predicting early clinical deterioration in patients hospitalized with suspected infection in a resource-constrained area in Southeast Asia. These findings could have critical implications for clinical care guidelines in similar areas where infection-related hospitalization and sepsis are common.

## Supplementary Material

ofae245_Supplementary_Data

## Supplementary Data

Supplementary materials are available at *Open Forum Infectious Diseases* online. Consisting of data provided by the authors to benefit the reader, the posted materials are not copyedited and are the sole responsibility of the authors, so questions or comments should be addressed to the corresponding author.

## References

[1] Rudd KE, Johnson SC, Agesa KM, et al Global, regional, and national sepsis incidence and mortality, 1990–2017: analysis for the Global Burden of Disease study. Lancet 2020; 6736:1–12. 10.1016/S0140-6736(19)32989-7 PMC6970225 31954465

[2] Rudd KE, Kissoon N, Limmathurotsakul D, et al The global burden of sepsis: barriers and potential solutions. Crit Care 2018; 22:1–11. 30243300 10.1186/s13054-018-2157-z PMC6151187

[3] Sudarmono P, Aman AT, Arif M, et al Causes and outcomes of sepsis in Southeast Asia: a multinational multicentre cross-sectional study. Lancet Glob Health 2017; 5:e157–67. 28104185 10.1016/S2214-109X(17)30007-4 PMC5332551

[4] Subbe CP, Kruger M, Rutherford P, Gemmel L. Validation of a modified Early Warning Score in medical admissions. QJM 2001; 94:521–6. 11588210 10.1093/qjmed/94.10.521

[5] Smith GB, Prytherch DR, Meredith P, Schmidt PE, Featherstone PI. The ability of the National Early Warning Score (NEWS) to discriminate patients at risk of early cardiac arrest, unanticipated intensive care unit admission, and death. Resuscitation 2013; 84:465–70. 23295778 10.1016/j.resuscitation.2012.12.016

[6] Hughes C, Pain C, Braithwaite J, Hillman K. “Between the flags”: implementing a rapid response system at scale. BMJ Qual Saf 2014; 23:714–7. 10.1136/bmjqs-2014-002845 24740239

[7] Pain C, Green M, Duff C, et al Between the flags: implementing a safety-net system at scale to recognise and manage deteriorating patients in the New South Wales public health system. Int J Qual Health Care 2017; 29:130–6. 27920243 10.1093/intqhc/mzw132

[8] Seymour CW, Liu VX, Iwashyna TJ, et al Assessment of clinical criteria for sepsis for the Third International Consensus Definitions for Sepsis and Septic Shock (Sepsis-3). JAMA 2016; 315:762–74. 26903335 10.1001/jama.2016.0288 PMC5433435

[9] Yu SC, Shivakumar N, Betthauser K, et al Comparison of early warning scores for sepsis early identification and prediction in the general ward setting. JAMIA Open 2021; 4:ooab062. 34820600 10.1093/jamiaopen/ooab062 PMC8607822

[10] Evans L, Rhodes A, Alhazzani W, et al Surviving sepsis campaign: international guidelines for management of sepsis and septic shock 2021. Intensive Care Med 2021; 47:1181–247. 34599691 10.1007/s00134-021-06506-y PMC8486643

[11] Liu VX, Lu Y, Carey KA, et al Comparison of early warning scoring systems for hospitalized patients with and without infection at risk for in-hospital mortality and transfer to the intensive care unit. JAMA Netw Open 2020; 3:e205191. 32427324 10.1001/jamanetworkopen.2020.5191 PMC7237982

[12] Churpek MM, Snyder A, Han X, et al Quick sepsis-related organ failure assessment, systemic inflammatory response syndrome, and early warning scores for detecting clinical deterioration in infected patients outside the intensive care unit. Am J Respir Crit Care Med 2017; 195:906–11. 27649072 10.1164/rccm.201604-0854OC PMC5387705

[13] Reuland C, Shi G, Deatras M, Ang M, Evangelista PPG, Shilkofski N. A qualitative study of barriers and facilitators to Pediatric Early Warning Score (PEWS) implementation in a resource-limited setting. Front Pediatr 2023; 11:1127752. 37009287 10.3389/fped.2023.1127752 PMC10050749

[14] Agulnik A, Ferrara G, Puerto-Torres M, et al Assessment of barriers and enablers to implementation of a pediatric early warning system in resource-limited settings. JAMA Netw Open 2022; 5:E221547. 35262714 10.1001/jamanetworkopen.2022.1547 PMC8908074

[15] Wheeler I, Price C, Sitch A, et al Early warning scores generated in developed healthcare settings are not sufficient at predicting early mortality in Blantyre, Malawi: a prospective cohort study. PloS One 2013; 8:e59830. 23555796 10.1371/journal.pone.0059830 PMC3612104

[16] Royal College of Physicians . National Early Warning Score (NEWS): standardising the assessment of acute-illness severity in the NHS. Report of a working party. RCP; 2012.

[17] Royal College of Physicians . National Early Warning Score (NEWS) 2: standardising the assessment of acute-illness severity in the NHS. Updated report of a working party. RCP; 2017.

[18] Hantrakun V, Somayaji R, Teparrukkul P, et al Clinical epidemiology and outcomes of community acquired infection and sepsis among hospitalized patients in a resource limited setting in Northeast Thailand: a prospective observational study (Ubon-sepsis). PloS One 2018; 13:1–14. 10.1371/journal.pone.0204509 PMC6157894 30256845

[19] Dellinger RP, Levy M, Rhodes A, et al Surviving sepsis campaign: international guidelines for management of severe sepsis and septic shock, 2012. Intensive Care Med 2013; 39:165–228. 23361625 10.1007/s00134-012-2769-8 PMC7095153

[20] Kelly A, Upex C, Bateman A, Nicholas Bateman D. Comparison of consciousness level assessment in the poisoned patient using the alert/verbal/painful/unresponsive scale and the Glasgow Coma Scale. Ann Emerg Med 2004; 44:108–13. 15278081 10.1016/j.annemergmed.2004.03.028

[21] Green M, Lander H, Snyder A, Hudson P, Churpek M, Edelson D. Comparison of the between the flags calling criteria to the MEWS, NEWS and the electronic Cardiac Arrest Risk Triage (eCART) score for the identification of deteriorating ward patients. Resuscitation 2018; 123:86–91. 29169912 10.1016/j.resuscitation.2017.10.028 PMC6556215

[22] Rudd KE, Seymour CW, Aluisio AR, et al Association of the quick Sequential (Sepsis-Related) Organ Failure Assessment (qSOFA) score with excess hospital mortality in adults with suspected infection in low- and middle-income countries. JAMA 2018; 319:2202–11. 29800114 10.1001/jama.2018.6229 PMC6134436

[23] Steyerberg EW, Harrell FE, Borsboom GJJM, Eijkemans MJC, Vergouwe Y, Habbema JDF. Internal validation of predictive models: efficiency of some procedures for logistic regression analysis. J Clin Epidemiol 2001; 54:774–81. 11470385 10.1016/s0895-4356(01)00341-9

[24] Fluss R, Faraggi D, Reiser B. Estimation of the Youden Index and its associated cutoff point. Biom J 2005; 47:458–72. 16161804 10.1002/bimj.200410135

[25] Collins GS, Reitsma JB, Altman DG, Moons KGM. Transparent reporting of a multivariable prediction model for individual prognosis or diagnosis (TRIPOD): the TRIPOD statement. BMC Med 2015; 13:1–10. 25563062 10.1186/s12916-014-0241-z PMC4284921

[26] Nakitende I, Nabiryo J, Namujwiga T, Wasingya-Kasereka L, Kellett J. Do different patient populations need different early warning scores? the performance of nine different early warning scores used on acutely ill patients admitted to a low-resource hospital in sub-Saharan Africa. Clin Med 2020; 20:1–7. 10.7861/clinmed.2019-0196 PMC6964175 31704729

[27] Pairattanakorn P, Angkasekwinai N, Sirijatuphat R, Wangchinda W, Tancharoen L, Thamlikitkul V. Diagnostic and prognostic utility compared among different sepsis scoring systems in adult patients with sepsis in Thailand: a prospective cohort study. Open Forum Infect Dis 2021; 8:ofaa573. 33447637 10.1093/ofid/ofaa573 PMC7781453

[28] Machado FR, Cavalcanti AB, Monteiro MB, et al Predictive accuracy of the Quick Sepsis-related Organ Failure Assessment score in Brazil a prospective multicenter study. Am J Respir Crit Care Med 2020; 201:789–98. 31910037 10.1164/rccm.201905-0917OC PMC7124712

[29] Wright SW, Hantrakun V, Rudd KE, et al Enhanced bedside mortality prediction combining point-of- care lactate and the quick Sequential Organ Failure Assessment (qSOFA) score in patients hospitalized with suspected infection in Southeast Asia : a cohort study. Lancet Glob Health 2022; 10:e1281–8. 35961351 10.1016/S2214-109X(22)00277-7 PMC9427027

[30] Haegdorens F, Monsieurs KG, De Meester K, Van Bogaert P. An intervention including the national early warning score improves patient monitoring practice and reduces mortality: a cluster randomized controlled trial. J Adv Nurs 2019; 75:1996–2005. 31012124 10.1111/jan.14034

[31] Booraphun S, Hantrakun V, Siriboon S, et al Effectiveness of a sepsis programme in a resource-limited setting: a retrospective analysis of data of a prospective observational study (Ubon-sepsis). BMJ Open 2021; 11:e041022. 10.1136/bmjopen-2020-041022 PMC7896572 33602702

[32] Thailand - Northeast Economic Development Report. World Bank Group; **2005**; 1–199. http://documents.worldbank.org/curated/en/116101468118461511/Thailand-Northeast-economic-development-report.

[33] Schultz MJ, Dunser MW, Dondorp AM, et al Current challenges in the management of sepsis in ICUs in resource-poor settings and suggestions for the future. Intensive Care Med 2017; 43:612–24. 28349179 10.1007/s00134-017-4750-z

[34] Ranjit S, Kissoon N. Challenges and solutions in translating sepsis guidelines into practice in resource-limited settings. Transl Pediatr 2021; 10:2646–65. 34765491 10.21037/tp-20-310 PMC8578780

[35] Brotherton BJ, Lelei F, Rudd KE. Clinical severity prediction scores in low-resource settings and the conundrum of missing data. JAMA Netw Open 2021; 4:e2137593. 34913984 10.1001/jamanetworkopen.2021.37593 PMC8730348

