# Supplementary material for: Prognostic Accuracy of Screening Tools for Clinical Deterioration in Adults With Suspected Sepsis in Northeastern Thailand: A Cohort Validation Study
Source: Open Forum Infect Dis. 2024 May 2;11(5):ofae245. doi: 10.1093/ofid/ofae245 (PMC11097208; doi:10.1093/ofid/ofae245)
Supplement: ofae245_Supplementary_Data [file ofae245_supplementary_data.pdf]

Prognostic accuracy of screening tools for clinical deterioration in adults with suspected sepsis in northeastern Thailand: a cohort validation study

Supplementary information

Contents (Page):

Supplementary Table 1: Enrollment systemic manifestation of infection criteria used for subject screening (2)
Supplementary Table 2: Calculated clinical prediction tool point systems (3)
Supplementary Table 3: Data availability (4)
Supplementary Table 4: Sensitivity analyses (5)
Supplementary Figure 1: Distribution of clinical deterioration outcomes (6)
Supplementary Figure 2: Distribution of patients and clinical deterioration by score (7-8)

Supplementary Table 1: Enrollment systemic manifestation of infection criteria used for subject screening

**Screening parameters**[a]	
1.	Body temperature > 38.3°C or < 36.0°C
2.	Heart rate > 90 beats per minute
3.	Respiratory rate > 20 per minute
4.	Glasgow Coma Score (GCS) < 15
5.	Plasma glucose > 140 mg/dL in the absence of diabetes
6.	White blood cell count > 12,000/µL or < 4000/µL or immature forms > 10%
7.	Plasma C-reactive protein >2 standard deviations above the normal value
8.	Plasma procalcitonin >2 standard deviations above the normal value
9.	Systolic blood pressure < 90 mmHg, mean arterial pressure < 70 mmHg, or SBP decrease > 40 mmHg
10.	SpO2 <95%
11.	PaO2 / FIO2 < 300
12.	Urine output < 0.5 mL/kg/hr or 45 mmol/L for 2 hours
13.	Creatinine increase > 0.5 mg/dL
14.	International normalized ratio >1.5 or activated partial thromboplastin time >60 seconds
15.	Platelet count < cells 100,000/µL
16.	Plasma total bilirubin > 4 mg/dL
17.	Lactate > 1 mmol/L
18.	Ileus defined by absence of bowel sounds documented on physical exam
19.	Decreased capillary refill or mottling documented on physical exam
20.	Significant edema documented on physical exam

[a] Screening parameters at enrollment for original cohort. Additional information can be found at: Hantrakun V, Somayaji R, Teparrukkul P, Boonsri C, Rudd K, Day NPJ, et al. Clinical epidemiology and outcomes of community acquired infection and sepsis among hospitalized patients in a resource limited setting in Northeast Thailand: A prospective observational study (Ubon-sepsis). PLoS One. 2018;13(9):1–14.

Supplementary Table 2: Calculated clinical prediction tool point systems

<table>
<tr><th>Component</th><th colspan="2">NEWS</th><th colspan="2">MEWS</th><th colspan="2">BTF[a]</th><th colspan="2">qSOFA</th></tr>
<tr><td rowspan="4">Respiratory rate (breaths/min)</td><td>≥25 or ≤8</td><td>3</td><td>≥30</td><td>3</td><td>≤5 or ≥30</td><td>2</td><td rowspan="2">≥22</td><td rowspan="2">1</td></tr>
<tr><td>21-24</td><td>2</td><td>≤9 or 21-29</td><td>2</td><td>6-10 or 25-29</td><td>1</td></tr>
<tr><td>9-11</td><td>1</td><td>15-20</td><td>1</td><td rowspan="2">11-24</td><td rowspan="2">0</td><td rowspan="2"><22</td><td rowspan="2">0</td></tr>
<tr><td>12-20</td><td>0</td><td>9-14</td><td>0</td></tr>
<tr><td rowspan="4">Temperature (°C)</td><td>≤35</td><td>3</td><td rowspan="2">≥38.5 or <35</td><td rowspan="2">2</td><td colspan="2" rowspan="4">-</td><td colspan="2" rowspan="4">-</td></tr>
<tr><td>>39</td><td>2</td></tr>
<tr><td>38.1-39 or 35.1-36</td><td>1</td><td rowspan="2">35-38.4</td><td rowspan="2">0</td></tr>
<tr><td>36.1-38</td><td>0</td></tr>
<tr><td rowspan="4">Systolic BP (mmHg)</td><td>≤90 or ≥220</td><td>3</td><td>≤70</td><td>3</td><td>≥200 or ≤90</td><td>2</td><td rowspan="2">≤100</td><td rowspan="2">1</td></tr>
<tr><td>91-100</td><td>2</td><td>71-80 or ≥200</td><td>2</td><td>180-199 or 91-99</td><td>1</td></tr>
<tr><td>101-110</td><td>1</td><td>81-100</td><td>1</td><td rowspan="2">100-179</td><td rowspan="2">0</td><td rowspan="2">>101</td><td rowspan="2">0</td></tr>
<tr><td>111-219</td><td>0</td><td>101-199</td><td>0</td></tr>
<tr><td rowspan="4">Heart rate (beats/min)</td><td>≥131 or ≤40</td><td>3</td><td>≥130</td><td>3</td><td>≥140 or ≤40</td><td>2</td><td colspan="2" rowspan="4">-</td></tr>
<tr><td>111-130</td><td>2</td><td>≤40 or 111-129</td><td>2</td><td>120-139 or 41-50</td><td>1</td></tr>
<tr><td>41-50 or 91-110</td><td>1</td><td>41-50 or 101-110</td><td>1</td><td rowspan="2">51-119</td><td rowspan="2">0</td></tr>
<tr><td>51-90</td><td>0</td><td>51-100</td><td>0</td></tr>
<tr><td rowspan="3">Level of consciousness[b]</td><td>V,P,U (GCS≤13)</td><td>3</td><td>P,U (GCS≤8)</td><td>3</td><td>P,U (GCS≤8)</td><td>2</td><td>GCS ≤14</td><td>1</td></tr>
<tr><td rowspan="2">A (GCS≥14)</td><td rowspan="2">0</td><td>V (GCS=9-13)</td><td>1</td><td>V (GCS=9-13)</td><td>1</td><td rowspan="2">GCS=15</td><td rowspan="2">0</td></tr>
<tr><td>A (GCS≥14)</td><td>0</td><td>A (GCS≥14)</td><td>0</td></tr>
<tr><td rowspan="2">Supplemental O_2</td><td>Yes</td><td>2</td><td colspan="2" rowspan="2">-</td><td colspan="2" rowspan="2">-</td><td colspan="2" rowspan="2">-</td></tr>
<tr><td>No</td><td>0</td></tr>
<tr><td rowspan="3">SpO_2</td><td>≤91%</td><td>3</td><td colspan="2" rowspan="3">-</td><td>≤90%</td><td>2</td><td colspan="2" rowspan="3">-</td></tr>
<tr><td>92-93%</td><td>2</td><td>91-95%</td><td>1</td></tr>
<tr><td>94-95%</td><td>1</td><td>≥96%</td><td>0</td></tr>
<tr><td>Range of possible values</td><td colspan="2">0-20</td><td colspan="2">0-14</td><td colspan="2">0-10</td><td colspan="2">0-3</td></tr>
</table>

[a] BTF uses a categorical Yellow & Red system rather than a numerical score. For the purposes of score calculation, Red=2, Yellow=1, Low risk=0.

[b] Level of consciousness for NEWS, MEWS and BTF are calculated using the AVPU system (Alert or responsive to Verbal stimuli, Painful stimuli or Unresponsive). As a Glasgow Coma Scale (GCS) was available at the time of score calculation, a GCS equivalent was used for score calculation.

Supplementary Table 3: Data availability at enrollment

Variable, total available (%)	Cohort (N=2680)
**Clinical deterioration outcome**	2680 (100)
**Baseline risk factors**	
Sex	2680 (100)
Age	2680 (100)
Pre-existing condition data	2680 (100)
Referral status	2680 (100)
**Score component**	
Respiratory rate	2662 (99.3)
Temperature	2675 (99.8)
Systolic blood pressure	2129 (79.4)
Heart rate	2678 (99.9)
Mental status assessment	2680 (100)
Supplemental oxygen	2678 (99.9)
SpO_2	2661 (99.3)

Supplementary Table 4: Sensitivity analyses

Sensitivity analysis	Clinical deterioration	Cohort AUROC (95% CI)	P value	Cross-validation AUROC (95% CI)[e]
**Complete cases[a] (N=2103)**	N=89/2103 (4%)			
**NEWS**		0.76 (0.72-0.80)	ref	0.75 (0.71-0.80)
**MEWS**		0.67 (0.62-0.72)	0.0004	0.65 (0.59-0.71)
**qSOFA**		0.63 (0.57-0.68)	<0.0001	0.60 (0.54-0.65)
**BTF**		0.68 (0.62-0.73)	0.005	0.65 (0.59-0.72)
**Prior value if missing[b] (N=2680)**	N=100/2680 (4%)			
**NEWS**		0.78 (0.74-0.82)	ref	0.77 (0.73-0.81)
**MEWS**		0.67 (0.62-0.72)	<0.0001	0.66 (0.60-0.71)
**qSOFA**		0.64 (0.59-0.69)	<0.0001	0.61 (0.55-0.66)
**BTF**		0.69 (0.63-0.74)	0.0006	0.67 (0.61-0.72)
**Admit-to-enrollment[c] (N=2680)**	N=100/2680 (4%)			
**NEWS**		0.77 (0.72-0.81)	ref	0.76 (0.71-0.80)
**MEWS**		0.64 (0.58-0.69)	<0.0001	0.63 (0.57-0.68)
**qSOFA**		0.67 (0.63-0.72)	<0.0001	0.63 (0.58-0.69)
**BTF**		0.67 (0.62-0.72)	<0.0001	0.65 (0.60-0.70)
**24±8 hours follow-up[d] (N=2930)**	N=124/2930 (4%)			
**NEWS**		0.79 (0.75-0.83)	ref	0.78 (0.74-0.82)
**MEWS**		0.68 (0.64-0.73)	<0.0001	0.66 (0.61-0.71)
**qSOFA**		0.66 (0.62-0.71)	<0.0001	0.62 (0.57-0.67)
**BTF**		0.69 (0.65-0.74)	0.0001	0.66 (0.61-0.71)

[a] Sensitivity analysis including only complete cases without missing variable data for score calculation at the time of enrollment.
[b] Sensitivity analysis including all subjects in the analysis cohort; when a score value was missing at enrollment, the most recently available prior value was used. In this analysis, all variables analyzed had >99% availability.
[c] Sensitivity analysis including clinical data available from admission to enrollment, all within 24 hours of admission. When a variable was available at both admission and enrollment, the worst variable according to score was selected. In this analysis, all variables analyzed had >99% availability.
[d] Sensitivity analysis for patients with follow-up data for clinical deterioration at 16-32 hours after enrollment rather than the 20-28 hour window used in the primary analysis.
[e] 10-fold internal cross-validation

Supplementary Figure 1: Distribution of clinical deterioration outcomes

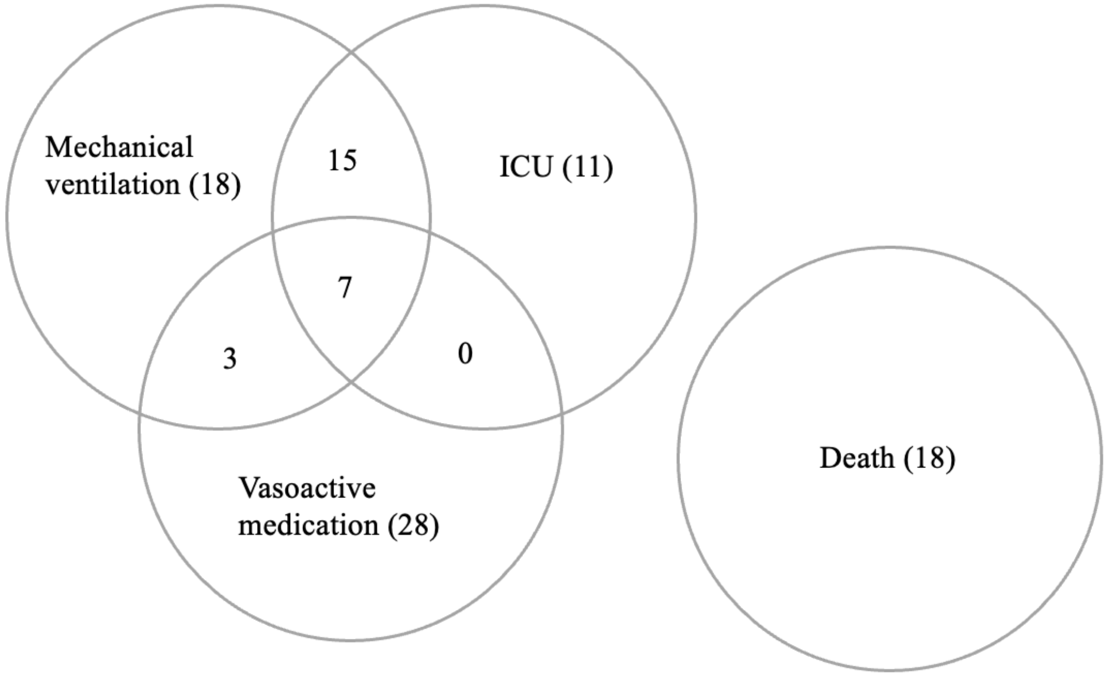

Venn diagram depicting the distribution of outcomes among the patients who experienced clinical deterioration in the primary analysis. Of the patients who clinically deteriorated, some received mechanical ventilation, some were started on vasoactive medications, some transferred to the ICU, and some experienced multiple of these clinical outcomes. These clinical details were not all available for the 18 patients who died within the follow-up window.

Supplementary Figure 2: Distribution of patients and clinical deterioration by score

A. NEWS

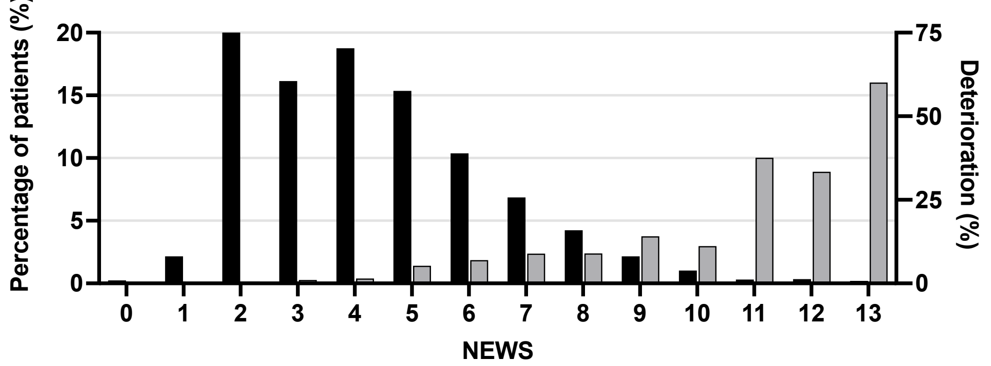

Number of patients	6	57	577	426	495	405	274	181	112	57	27	8	9	5

B. MEWS

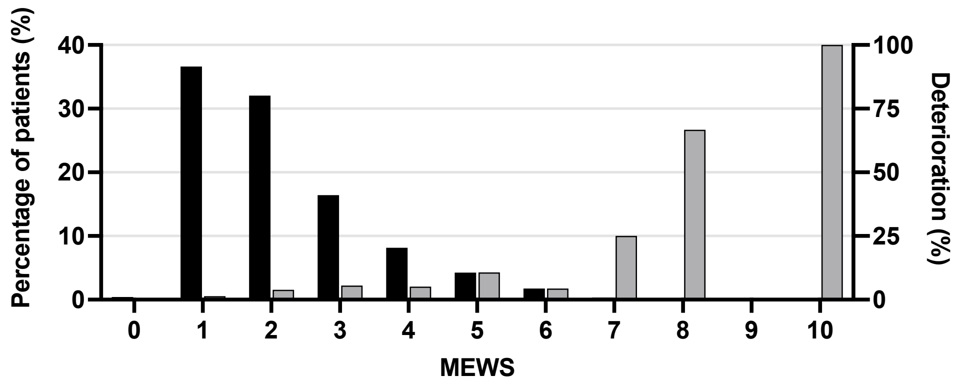

Number of patients	10	927	846	433	215	112	46	8	3	0	1

C. qSOFA

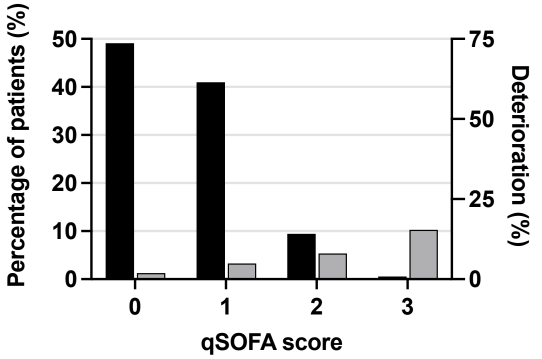

Number of patients	1296	1081	249	13

D. Between the Flags

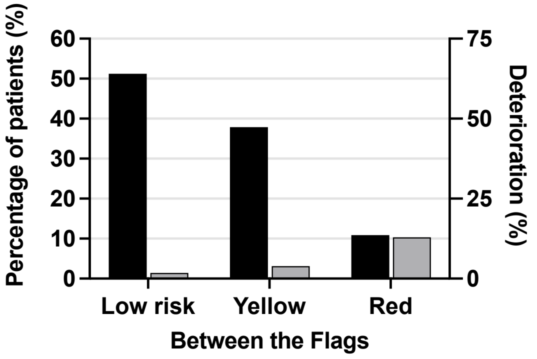

Number of patients	1352	1000	287

Bars show distribution of patients (% of cohort, black bars, left Y-axis) and clinical deterioration (% of patients within each score value, gray bars, right Y-axis) by NEWS (A), MEWS (B), qSOFA (C) and BTF (D). Abbreviations: NEWS (National Early Warning Score), MEWS (Modified Early Warning Score), qSOFA (quick Sequential Organ Failure Assessment), BTF (Between the Flags)
